# Distinct Phospho-TDP-43 brain distribution in two cases of FTD, one associated with ALS

**DOI:** 10.1590/1980-57642016dn11-030006

**Published:** 2017

**Authors:** Álvaro C.B. Guedes, Ricardo Santin, André S.R. Costa, Keli C. Reiter, Arlete Hilbig, Liana L. Fernandez

**Affiliations:** 1 Students at the Medical School of Federal University of Health Sciences of Porto Alegre. Scientific initiation scholars.; 2 Biologist, PhD, Federal University of Health Science of Porto Alegre's Laboratory of Pathology.; 3 Neurologists. MD, PhD, Professors at the Federal University of Health Science of Porto Alegre.

**Keywords:** TDP-43, frontotemporal dementia, amyotrophic lateral sclerosis, neuropathology, TDP-43, demência frontotemporal, esclerose lateral amiotrófica, neuropatologia

## Abstract

**INTRODUCTION:**

TDP-43 is an intranuclear protein involved in many cellular processes. When altered, it shows a change in pattern of distribution, as well as in functioning, throughout the Central Nervous System structures. Frontotemporal Lobar Degeneration (FTLD) and Amyotrophic Lateral Sclerosis (ALS) are examples of TDP-43 proteinopathy. These disorders form a clinical spectrum, with some patients having a pure cognitive disorder while others also exhibit motor features.

**METHODS:**

We studied two donated brains from patients with a diagnosis of Frontotemporal Dementia (FTD), one of which was associated with ALS (ALS-FTD). After fixation and macroscopic examinations, sample analyses were performed. Specific regions were chosen for the application of immunohistochemistry (IHC) with anti-Aβ, AT8, anti-α-synuclein and anti-phospho-TDP-43.

**RESULTS:**

Both brains presented anti-phospho-TDP-43 positivity, but this was not equally distributed throughout the encephalic zones. In the FTD case, the studied brain presented phosphorylated TDP-43- in the frontal cortex, hippocampus, entorhinal cortex and mesencephalon; in the ALS-FTD case, the abnormal protein was also seen in the pons and medulla oblongata. The brain in the ALS-FTD case presented Aβ and AT8 positivity in the hippocampus and entorhinal cortex (Braak I and II).

**DISCUSSION:**

The hypothesis supported by scientific literature that these neurodegenerative diseases can have the same etiology with distinct encephalic region involvement is corroborated by the present study.

## RESUMO

**INTRODUÇÃO:**

TDP-43 é uma proteína intranuclear envolvida em vários processos celulares. Essa molécula, quando alterada, mostra padrões de distribuição modificados, assim como de funcionamento, ao longo das estruturas do Sistema Nervoso Central. A Degeneração Lobar Frontotemporal (DLFT) e a Esclerose Lateral Amiotrófica (ELA) são dois exemplos de proteinopatia de TDP-43. Esses transtornos formam um espectro clínico, com alguns pacientes apresentando um transtorno cognitivo puro enquanto outros também apresentam disfunções motoras.

**MÉTODOS:**

Nós estudamos dois cérebros doados de pacientes com diagnóstico de Demência Frontotemporal (DFT), um dos quais se associava com ELA (ELA-DFT). Após fixação e exame macroscópico, foram realizadas análises de amostras. Regiões específicas foram escolhidas para aplicação de imunohistoquímica (IHQ) com anti-Aβ, AT8, anti-α-sinucleina e anti-fosfo-TDP-43.

**RESULTADOS:**

Ambos os cérebros foram positivos para anti-fosfo-TDP-43, mas de forma não igualmente distribuida pelas regiões encefálicas. No caso DFT, o cérebro estudado apresentou TDP-43-fosforilada no córtex frontal, hipocampo, córtex entorrinal e mesencéfalo; no caso ELA-DFT, a proteína anormal também foi vista na ponte e no bulbo. O cérebro do caso ELA-DFT foi positivo para Aβ e AT8 no hipocampo e no córtex entorrinal (Braak I e II).

**DISCUSSÃO:**

O presente estudo corrobora a hipótese atualmente sustentada pela literatura científica de que essas duas doenças neurodegenerativas possuem a mesma etiologia, mas acometem regiões encefálicas distintas.

## INTRODUCTION

Neurodegenerative diseases present some cardinal features, for instance: they affect specific systems or functional parts of the nervous system and usually have an insidious onset, appearing after a long period of normal functioning of the nervous system, and have a relentless progressive course.^1^ Another characteristic is that they form deposits of proteins that change their conformation, becoming anomalous.^2^

Frontotemporal Lobar Degeneration (FTLD) is the term used to describe a group of neurodegenerative disorders with predominant involvement of frontal and temporal lobes.^3^ It is a pathological and genetically heterogeneous condition^4^ associated with the clinical entity of frontotemporal dementia (FTD) which represents one of the most common neurodegenerative disorders, generally having an early onset and impairment of cognition, language or behavior.^5^ Histologically, almost half of such cases have tau-based pathology, half TDP-43-based pathology, and about 5% have FUS-based pathology.^6^

Amyotrophic Lateral Sclerosis (ALS) is also a neurodegenerative disease. Its signs, however, are due to progressive loss of motor neurons – both the upper motor neuron (UMN) and the lower motor neuron (LMN)^7^ – which typically results in progressive muscle weakness, leading to palsy and death within three to five years.^8^ When degeneration affects motor neurons in the medulla oblongata, in the brain stem, dysarthria and dysphagia can be some of the early manifestations of the disease.

The transactive response (TAR) DNA-binding protein with an M_r_ of 43 kD (TDP-43), whose gene is found on chromosome one,^9^ is an intranuclear protein involved in different cellular processes such as gene transcription, alternative splicing, mRNA stability, microRNA biogenesis, cell division and apoptosis. When altered, this molecule changes its pattern of distribution and function throughout the CNS structures.

FTD and ALS are two examples of TDP-43-proteinopathies. Having clinically different presentation from each other, FTD and ALS were usually assessed by different neurological researchers. However, a growing body of evidence has been collected over the last decade, leading researchers and clinicians to believe that these two entities are, in fact, two sides of the same coin, sharing a common pathological basis.^8^^,^ 10 ^-^ 14

The present study describes the phospho-TDP-43 immunohistochemical reaction (IHC) distribution in encephalic regions of two patients with clinical features of FTD, one of which had motor involvement consistent with ALS.

## METHODS

This is a descriptive study of the samples of two donated brains from patients who had been monitored by the Clinical Neurology staff of the ISCMPA (Southern Brazil), after informed consent was obtained from first-degree relatives. The postmortem interval was less than 24 hours. The donors' clinical information was obtained from the next-of-kin and from the medical specialist records kept when the patients were in the hospital. After removal, the brains were fixed in 10% formalin solution for four weeks; one hemisphere (the left) was sectioned, and macroscopically evaluated, in accordance with the international protocol. Frontal (inferior and superior levels), parietal, temporal (inferior and superior levels), occipital, hippocampus (at the level of the mammillary body and at the level of the lateral geniculate body), amygdala, entorhinal cortex, mesencephalon, pons and medulla oblongata were sampled for histopathological evaluation and submitted to the IHC technique. The IHC technique was performed in compliance with the routine protocols of the Laboratory of Pathology at the UFCSPA. After deparaffinization, the samples were immersed in 3% hydrogen peroxide and 10% methanol for 15 minutes to inhibit endogenous peroxidase activity. The sample were then boiled (92ºC) in citrate (10 mM, pH 6.0) and/or treated with 1% formic acid to recover the antigen. After washing with PBS, they were incubated with normal horse serum for 1 hour and then with primary antibody at 4ºC in a wet chamber overnight. The following primary antibodies were used: anti-Aβ (human-mouse monoclonal antibody, DAKOCYTOMATION, clone 6F/3D, code M0872), 1:25 dilution, following 3 minutes of incubation in 1% formic acid; anti-phosphorylated tau (monoclonal mouse antibody, INNOGENETICS, clone AT-8, code 90206), 1:500 dilution, following 10 minutes of incubation in citrate; and anti-alpha-synuclein (mouse monoclonal antibody, NOVOCASTRA, clone KM51, code ASYN-L), 1:200 dilution, following 4 minutes of incubation in 1% formic acid and 20 minutes of incubation in citrate and anti-phospho-TDP-43 (COSMO BIO CO, Tip-PDT-P05), dilution 1:2500, pretreated with citrate for 20 minutes. After overnight incubation in primary antibodies, the slides were washed three times in PBS and incubated in DAKO secondary polymer for 40 minutes, Streptavidin HRO, DAKO for 30 minutes, and finally treated with DAB, Sigma, for 3 minutes. All of the slides were counterstained with hematoxylin for 10 seconds. All of the procedures were performed with negative and positive controls. Slides were evaluated with a light microscope where protein deposits were searched by three independent observers and photos taken. The analysis was performed with an Olympus BX51 microscope using a high magnification objective (10x, 20x, 40x). The tissue images were captured using a high-resolution digital camera (DP-72) attached to the microscope. Neuropathological findings were compared with the clinical manifestations of patients and discussed.

The UFCSPA ethics committees approved the study.

## RESULTS

### Clinical description.

*Case 1 (ALS-FTD):* 54-year-old man, truck driver, initially presented with dysarthria and dysphagia the previous year. He progressively developed mood swings and emotional lability, episodic memory loss with temporal and spatial disorientation, apraxia, and non-fluent aphasia with activities of daily living impairment. These symptoms were followed by weakness, first in the upper and then in the lower limbs, with muscular atrophy and fasciculation, bilateral Babinski and presence of frontal release signs, and cranial nerve IX and X deficits. Finally, he presented behavioral disorder becoming aggressive. After 2 years, he died from aspiration pneumonia followed by septicemia. He was a smoker and used alcohol. He had no family history of degenerative disorders. He was submitted to a laboratory investigation with negative polyclonal immunoglobulin test, to a skull MRI that demonstrated diffuse cortical atrophy, and to electromyography disclosing signs consistent with compromise of bulbar and spinal motor neurons. *Case 2 (FTD):* 55-year-old woman, retired, initially presented with insidious and progressive behavioral disorder characterized by isolation, loss of social interaction, and confusion with temporal and spatial disorientation. Her relatives inferred that she was depressive and first admitted to a psychiatric hospital and then transferred to a neurology clinic. She developed echolalia and activities of daily living impairment. She was submitted to a skull MRI that demonstrated fronto-temporal atrophy with frontal cortical gyros presenting hyperintensity (T2 and Flair) suggesting Pick disease. The exam also demonstrated volume reduction in the basal ganglia. She was also submitted to a lumbar puncture with normal CSF investigation. After 3 years she was aphasic, with temporal and spatial disorientation, developed akathisia and was completely dependent, including on gastrostomy feeding. She later presented lower limb dystonia and loss of bowel control and died from septicemia caused by a urinary tract infection. Her father had had dementia syndrome (not investigated) with the onset of symptoms at 70 years.

### Neuropathological description.

Both cases presented moderate to severe frontal and temporal atrophy, without asymmetry, according to macroscopic findings. The FTD case presented more ventricular enlargement and atrophy throughout basal ganglia (without significant difference between them). Substantia nigra and locus coeruleus pigmentation were normal in both cases.

Both cases presented superficial microvacuolation, especially in the FTD case, neuronal loss, and white matter involvement, particularly in the ALS-FDT case. These pathological findings were more prominent in the frontal area, but also occurred in the temporal area.

**Figure 1:**
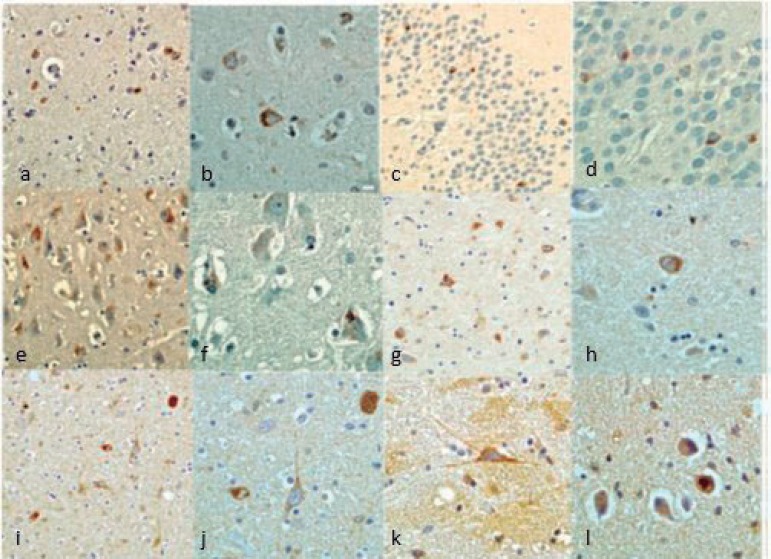
FTD/ALS (case 1) Phospho-TDP43 IHQ. [A] Frontal cortex (200×); [B] Frontal cortex (400×); [C] Hippocampus dentate granular cells (200×); [D] Hippocampus dentate granular cells (400×); [E] Entorhinal cortex (200×); [F] Entorhinal cortex (400×); [G] Mesencephalon (200×); [H] Mesencephalon (400×); [I] Pons (2000×); [J] Pons (400×); [K-L] Medulla olblongata (400×).

**Figure 2:**
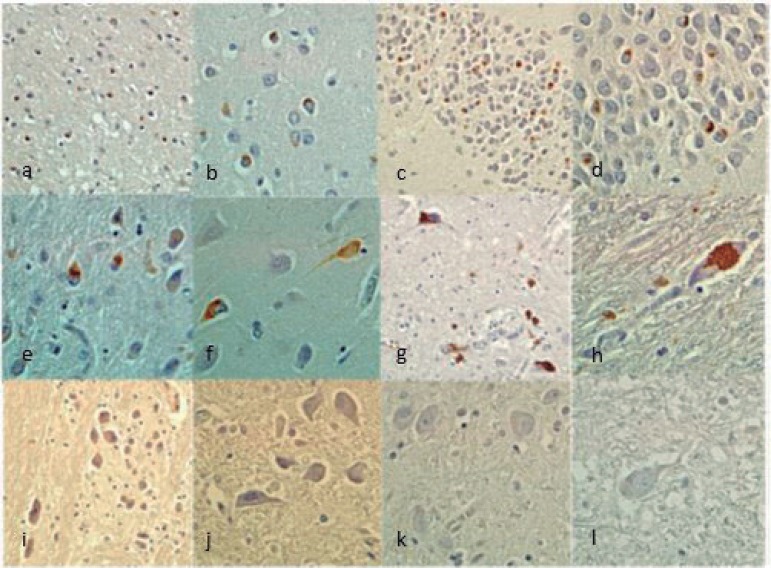
FTD (case 2) Phospho-TDP43 IHQ. [A] Frontal cortex (200×); [B] Frontal cortex (400×); [C] Hippocampus dentate granular cells (200×); [D] Hippocampus dentate granular cells (400×); [E] Entorhinal cortex (200×); [F] Entorhinal cortex (400×); [G] Mesencephalon (200×); [H] Mesencephalon (400×); [I] Pons (200×); [J] Pons (400×); [K-L] Medulla oblongata (400×).

The ALS-FTD case presented Aβ (Phase II of Thal staging and CERAD A criteria) and AT8 immunoreactivity in the hippocampus and entorhinal cortex considered stage I/II of Braak's Classification for Alzheimer's disease (AD).^15^ Both cases presented non-immunoreactivity for α-synuclein. Phospho-TDP-43 IHC was positive in both cases but with distinct distribution. The ALS-FTD case showed positive phospho-TDP-43 neuronal cytoplasmic inclusions (NCI) in all regions studied (frontal, parietal, temporal, occipital, amygdala, hippocampus, entorhinal cortex, mesencephalon, pons and medulla oblongata) (Figure 1), while the FTD case presented immunoreactivity in all regions except the pons and medulla oblongata (Figure 2). Both cases had a predominance of NCI over dystrophic neurites (DN) in layer II of the neocortex. However, the inclusions were found only in layer II in the FTD case, but were present in other layers in the ALS-FTD case. NCI in neocortex were more frequent in the FTD case. In the dentate granule cell layer of the hippocampus, the FTD case had moderate granular NCI but these were mild and compact in the ALS-FTD case. NCI in the entorhinal cortex were more frequent in the FTD case. Both cases presented oligodendroglial cytoplasmatic inclusions but these were more frequent in the ALS-FTD case. Granular cytoplasmatic inclusions were found only in the ALS-FTD case, markedly in neurons of the inferior olivary nucleus and hypoglossal nucleus, in medulla oblongata. Unfortunately, the spinal cord was not available to be submitted to IHC.

## DISCUSSION

The main feature of FTLD is progressive degeneration of the frontal and temporal lobes of the brain,^12^ observed in both our macroscopic brain examinations.

Of all clinical syndromes, the most frequent presentation of FTLD is FTD. Among FTD varieties, three main syndromes can be recognized: behavioral variant frontotemporal dementia (bv-FTD), which involves personality and behavioral changes; semantic dementia (SD) with fluent language alteration; or progressive nonfluent aphasia (PNFA), characterized by nonfluent language alteration.^12^^,^ 16 ^-^ 19 Despite this classification, there is a clinical, pathological, and genetic overlap. For instance, in advanced stages, SD cases may develop features of bv-FTD,^20^ and there is also an overlap between FTD and other neurodegenerative diseases such as progressive supranuclear palsy (PSP), corticobasal degeneration (CBD) and ALS.^21^^-^ 23 Notably, all three FTD syndromes can be accompanied by signs of ALS in up to 15% of patients, although bv-FTD-ALS is the most common combination.^6^ In case 1 (ALS-FTD), dysphagia and dysarthria were the first signs characterizing motor neurons of IX and X nuclear involvement in the brain stem followed by PNFA, upper and lower limbs, and, finally, behavioral alteration corroborating with this overlap.

Differentiating one variant of FTD from another, as well as from other neurodegenerative (for instance atypical AD) and psychiatric diseases, remains challenging.^21^ Case 2 (FTD) was diagnosed with bv-FTD, but this was initially thought to be depression, and with the progression of the disease language impairment developed.

Both cases presented an early onset dementia. Following Alzheimer's disease, FTD is the most common dementia in patients younger than 65 years old.^18^ In Brazil, epidemiological studies estimate that the average age for the onset of ALS is 53 to 54 years^24^^,^ 25 with a prevalence in Southern Brazil of 5 cases per 100,000 population.^26^

Much has been discovered about the involvement of proteins in neurodegenerative diseases, such as amyloid aggregates and defects in tau protein in Alzheimer's disease.^27^^,^ 28 For FTD, nearly 50% of its "behavioral variant" involve tauopathies or TDP-43-proteinopathies. Between 5-10% of these have FUS protein deposits (fused in sarcoma) and 70% of cases of PNFA show tau protein deposits, whereas 30% display accumulation of TDP-43 protein. More than 90% of SD cases exhibit TDP-43 deposits while less than 10% are tauopathies.^29^

Findings from the current and last decade reinforce the unifying hypothesis that put FTD and ALS in the group of the so-called proteinopathies by TDP-43, stating that TDP-43 abnormal accumulation is a shared molecular basis.^9^^,^ 31 Some of the known TDP-43 biochemical abnormalities include hyper-phosphorylation, ubiquitination, and truncation of its N-terminal portion. Although all TDP-43 functions remain unknown, it has been discovered that it is a ubiquitous protein and plays an important role in various cellular processes such as gene transcription, alternative splicing and stabilization of mRNA, microRNA biogenesis, apoptosis and cell division.^32^ In the CNS, TDP-43 can be normally found in neuron and glial cell nuclei; in pathological conditions, however, the protein accumulates in cytoplasm and in the cell axon. In such cases, abnormal TDP-43 is seen in its hyper-phosphorylated presentation. Consequently, IHC diagnosis of TDP-43-proteinopathies finds greater concordance among neuropathologists when the anti-phosphorylated-TDP-43 antibody has been applied.^33^^,^ 34

Based on pathological considerations, TDP-43 neural inclusions may be classified as follows: neuronal cytoplasmatic inclusions (NCI), dystrophic neurites (DN), neuronal intranuclear inclusions (NII) and glial cytoplasmic inclusions (GCI). It is possible to distinguish neuropathological subtypes of FTLD-TDP by the predominant type of inclusions. It is well known that the particular distribution of the pathology is more predictive of the clinical presentation than the molecular nature of the pathology.^30^ Both cases presented clinical signs of FTD characterized by behavioral disorder, non-fluent aphasia and apraxia and also had NCI of phospho-TDP-43 distributed in cortical and subcortical regions. However, the ALS-FTD case, which had motor signs and bulbar involvement with changes in speech and swallowing, presented NCI and DN in the pons and medulla oblongata with involvement of inferior olivary and hypoglossal nuclei. The brainstem was preserved in the FTD case, which was compatible with the clinical features.

The predominance of NCI over DN was observed in our cases, characterizing Mackenzie type 3.^10^^,^ 30 On the other hand, the FTD case presented NCI only in layer II which is a characteristic of Mackenzie type 1 while the ALS-FTD case also had inclusions in other layers, compatible with Mackenzie type 3.^10^^,^ 30 The granular and compact type NCI in dentate granule cells of the hippocampus is not able to distinguish these two pathological subtypes. Progranulin gene mutations are associated with Mackenzie type 1 and not with type 3. Thus, if this gene could be investigated, the correct classification would be elucidated.

ALS and FTD seem to form a clinical spectrum with some patients having a pure cognitive disorder, while others exhibit both motor and cognitive features.^13^ Many issues remain unresolved. The relationship between genetic, pathologic, and clinical phenotype is of the utmost importance, as it enables the identification of pathological subtypes *in vivo*. In the future, it is expected that biomarkers will be identified that allow a specific therapy to be tailored according to the underlying pathology.^21^

The degree of overlap between ALS and FTD syndromes is also a topic of current research interest. The hypothesis supported by scientific literature that these neurodegenerative diseases could have the same etiology yet distinct encephalic region involvement is corroborated by the present study.
